# Preoperative radiochemotherapy versus immediate surgery for resectable and borderline resectable pancreatic cancer (PREOPANC trial): study protocol for a multicentre randomized controlled trial

**DOI:** 10.1186/s13063-016-1262-z

**Published:** 2016-03-09

**Authors:** Eva Versteijne, Casper H. J. van Eijck, Cornelis J. A. Punt, Mustafa Suker, Aeilko H. Zwinderman, Miriam A. C. Dohmen, Karin B. C. Groothuis, Oliver R. C. Busch, Marc G. H. Besselink, Ignace H. J. T. de Hingh, Albert J. ten Tije, Gijs A. Patijn, Bert A. Bonsing, Judith de Vos-Geelen, Joost M. Klaase, Sebastiaan Festen, Djamila Boerma, Joris I. Erdmann, I. Quintus. Molenaar, Erwin van der Harst, Marion B. van der Kolk, Coen R. N. Rasch, Geertjan van Tienhoven, for the Dutch Pancreatic Cancer Group (DPCG)

**Affiliations:** Department of Radiation Oncology, Academic Medical Center, Meibergdreef 9, 1105 AZ Amsterdam, The Netherlands; Department of Surgery, Erasmus Medical Center, Postbus 2040, 3000 CA Rotterdam, The Netherlands; Department of Medical Oncology, Academic Medical Center, Meibergdreef 9, 1105 AZ Amsterdam, The Netherlands; Department of Clinical Epidemiologic Biostatics, Academic Medical Center, Meibergdreef 9, 1105 AZ Amsterdam, The Netherlands; Clinical Research Department, Comprehensive Cancer Organisation the Netherlands (IKNL), Postbus 1281, 6501 BG Nijmegen, The Netherlands; Department of Surgery, Academic Medical Center, Meibergdreef 9, 1105 AZ Amsterdam, The Netherlands; Department of Surgery, Catharina Hospital, Postbus 1350, 5602 ZA Eindhoven, The Netherlands; Department of Medical Oncology, Amphia Hospital, Postbus 90158, 4800 RK Breda, The Netherlands; Department of Surgery, Isala Clinics, Postbus 10400, 8000 GK Zwolle, The Netherlands; Department of Surgery, Leiden University Medical Center, Postbus 9600, 2300 RC Leiden, The Netherlands; Department of Medical Oncology, Maastricht University Medical Center, Postbus 3035, 6202 NA Maastricht, The Netherlands; Department of Surgery, Medical Spectrum Twente, Postbus 50 000, 7500 KA Enschede, The Netherlands; Department of Surgery, Onze Lieve Vrouwe Gasthuis, Postbus 95500, 1090 HM Amsterdam, The Netherlands; Department of Surgery, Sint Antonius Hospital, Postbus 2500, 3430 EM Nieuwegein, The Netherlands; Department of Surgery, University Medical Center Groningen, Postbus 30.001, 9700 RB Groningen, The Netherlands; Department of Surgery, University Medical Center Utrecht, Postbus 85500, 3508 GA Utrecht, The Netherlands; Department of Surgery, Maasstad Hospital, Maasstadweg 21, 3079 DZ Rotterdam, The Netherlands; Department of Surgery, Radboud University Medical Center, Geert Grooteplein-Zuid 10, 6525 GA Nijmegen, The Netherlands

**Keywords:** (Borderline) resectable pancreatic cancer, Preoperative radiochemotherapy, Explorative laparotomy, Overall survival, Intention to treat

## Abstract

**Background:**

Pancreatic cancer is the fourth largest cause of cancer death in the United States and Europe with over 100,000 deaths per year in Europe alone. The overall 5-year survival ranges from 2–7 % and has hardly improved over the last two decades. Approximately 15 % of all patients have resectable disease at diagnosis, and of those, only a subgroup has a resectable tumour at surgical exploration. Data from cohort studies have suggested that outcome can be improved by preoperative radiochemotherapy, but data from well-designed randomized studies are lacking. Our PREOPANC phase III trial aims to test the hypothesis that median overall survival of patients with resectable or borderline resectable pancreatic cancer can be improved with preoperative radiochemotherapy.

**Methods/design:**

The PREOPANC trial is a randomized, controlled, multicentric superiority trial, initiated by the Dutch Pancreatic Cancer Group. Patients with (borderline) resectable pancreatic cancer are randomized to A: direct explorative laparotomy or B: after negative diagnostic laparoscopy, preoperative radiochemotherapy, followed by explorative laparotomy. A hypofractionated radiation scheme of 15 fractions of 2.4 gray (Gy) is combined with a course of gemcitabine, 1,000 mg/m^2^/dose on days 1, 8 and 15, preceded and followed by a modified course of gemcitabine. The target volumes of radiation are delineated on a 4D CT scan, where at least 95 % of the prescribed dose of 36 Gy in 15 fractions should cover 98 % of the planning target volume. Standard adjuvant chemotherapy is administered in both treatment arms after resection (six cycles in arm A and four in arm B). In total, 244 patients will be randomized in 17 hospitals in the Netherlands. The primary endpoint is overall survival by intention to treat. Secondary endpoints are (R0) resection rate, disease-free survival, time to locoregional recurrence or distant metastases and perioperative complications. Secondary endpoints for the experimental arm are toxicity and radiologic and pathologic response.

**Discussion:**

The PREOPANC trial is designed to investigate whether preoperative radiochemotherapy improves overall survival by means of increased (R0) resection rates in patients with resectable or borderline resectable pancreatic cancer.

**Trial registration:**

Trial open for accrual: 3 April 2013

The Netherlands National Trial Register – NTR3709 (8 November 2012)

EU Clinical Trials Register – 2012-003181-40 (11 December 2012)

## Background

Pancreatic cancer is the fourth largest cause of cancer death in the United States and Europe with over 100,000 deaths per year in Europe alone [1, 2]. The overall 5-year survival ranges from 2–7 % and has hardly improved over the last two decades [1, 3, 4]. Surgery is the main treatment option that may lead to cure. After radical resection the median survival is about 15–20 months and the 5-year overall survival ranges from 8–25 % [5–8]. The outcome may be improved by preoperative radiochemotherapy, but data from well-designed randomized studies are lacking [9]. Recently, a monocentric randomized controlled trial on preoperative radiochemotherapy was terminated early because of poor recruitment [10]. Research should be focused on a coordinated multidisciplinary approach to improve results for patients with resectable and borderline resectable pancreatic cancer.

Pancreatoduodenectomy is the commonly accepted surgical treatment for resectable cancer of the pancreatic head. The goal of pancreatoduodenectomy is to perform a microscopically complete (R0) resection of the tumour. A microscopically incomplete resection (R1) worsens the prognosis [11–14]. The resection rate depends upon the preoperative radiologic workup with high quality CT scan, MRI and/or laparoscopy, and is reported as 60 % without and 82 % with laparoscopy [15]. The R0 resection rate varies from 25–84 % and depends upon the dissection technique and pathological workup [16].

In the 2014 Dutch National Audit 33 % of patients appeared to have an irresectable tumour at explorative laparotomy after radiologic assessment [17]. The most important prognostic factors are tumour size and vascular involvement; large tumours (>2 cm) have a worse prognosis than smaller tumours [11, 18]. Tumours ≤2 cm without vessel involvement, classified as T1 according to the UICC TNM classification, have the best chance of an R0 resection and may not benefit from preoperative radiochemotherapy. Encasements of the celiac axis, superior mesenteric artery (SMA) or common hepatic artery (CHA) are considered contraindications for resection. Resectability in the case of involvement of the portal vein and/or the superior mesenteric vein (PV/SMV) is under debate.

Surgical series vary in criteria for resectability. Series of patients undergoing curative surgery tend to focus on survival data of the patients who actually underwent a successful resection. Reporting data by intention to treat, that is, on all patients who were selected for explorative surgery, whether or not the resection was performed at all, would provide more relevant data. Data from studies reporting the survival of patient by intent to treat after preoperative therapy report a median overall survival (mOS) of 17 months [19–25]. Studies of explorative surgery without preoperative treatment analysing survival by intention to treat report mOS between 10 and 12 months [22, 26, 27]. These survival rates are not comparable with those of studies on postoperative adjuvant (radio)chemotherapy. In this last category, the median survival is better due to selection bias, since in these studies only patients who were fit enough after a successful resection participated. In the systematic review of Gillen et al., the actual resection rates are in the order of 70–85 % of patients who were considered to have a resectable tumour at preoperative workup (with or without preoperative radiochemotherapy). Reporting data by intention to treat seems advisable and would change outcome figures of surgical series significantly [9]. Therefore, future studies in patients with resectable pancreatic cancer should have a prospective randomized design, with analyses by intention to treat, to be able to investigate not only the improvement of the prognosis as such, but also the potential improvement of the resection rate.

The rationale for preoperative therapy in pancreatic cancer is multifold. First, radiochemotherapy administered before surgery to non-dissected, well-oxygenated tissue may maximize any potential benefit of both radiation and chemotherapy, as compared to postoperative radiochemotherapy. Second, preoperative radiochemotherapy may decrease tumour volume, thus improving resectability and minimize regional nodal disease, hence reducing the risk of loco-regional recurrence. Third, it may downstage disease by sterilizing the peripheral extent of tumour infiltration, resulting in an increased proportion of R0 resections. Randomized controlled trials investigating the effect of postoperative adjuvant radiochemotherapy were all negative [28–32]. A number of prospective, single arm, single centre studies suggested an improved resectability rate as well as a higher proportion of R0 resections by preoperative radiochemotherapy [19–23, 33–38]. Although the numbers of patients are too small for firm conclusions, the results of studies in patients with borderline resectable tumours who received preoperative treatment suggest that this strategy is not inferior and may even be better compared with patients who had a resection without preoperative treatment [22]. This was also the conclusion of a meta-analysis of Gillen et al., which included 111 studies of preoperative treatment, 56 of which were performed in patients with tumours that were initially considered irresectable. In these studies, of 147 patients with initially irresectable tumours, 33 % of patients underwent a successful resection after preoperative therapy. Remarkably, the R0 resection rate (79 %) and the median survival (20.5 months) in this group were similar to the results of studies in patients with primarily resectable tumours [9]. Artinyan et al. performed a population-based regional retrospective review of 354 patients with resected pancreatic cancer and observed a better median survival for preoperative therapy compared to postoperative adjuvant therapy (34 versus 19 months, respectively, HR 0.57, *p* = 0.013) [39]; keep in mind that these are different groups.

The hypothesis that preoperative radiochemotherapy may improve the outcome of resectable and borderline resectable pancreatic cancer is worth testing [40]. In a recent European consensus, preoperative radiochemotherapy was considered as one of the main directions for future clinical research [41]. Within the Dutch Pancreatic Cancer Group (DPCG), this concept has been further developed into the clinical randomized, controlled, multicentre randomized phase III PREOPANC trial. This trial tests the hypothesis that preoperative radiochemotherapy followed by explorative surgery compared to direct explorative surgery may improve the survival of patients with resectable or borderline resectable pancreatic cancer. This trial will be analysed by intention to treat.

## Methods/design

### Design

The PREOPANC trial is a randomized, controlled, multicentre trial, initiated by the DPCG. Patients with resectable or borderline resectable tumours (see Table 1) are randomized to arm A: direct explorative laparotomy or arm B: preoperative radiochemotherapy, followed by explorative laparotomy. In both arms patients receive standard adjuvant chemotherapy after resection.

**Table 1:** Dutch Pancreatic Cancer Group definitions for resectability of pancreatic adenocarcinoma (DPCG, 2012)

	SMA	Celiac axis	CHA	SMV-PV
Resectable (all four required)	no contact	no contact	no contact	≤90° contact
Borderline resectable (minimally one required)	≤90° contact	≤90° contact	≤90° contact	≤90°-270° contact, and no occlusion
Irresectable (minimally one required)	contact > 90°	contact > 90°	contact > 90°	contact > 270° or occlusion

*SMA* superior mesenteric artery, *CHA* common hepatic artery, *SMV* superior mesenteric vein, *PV* portal vein

The aim of the study is to investigate whether preoperative radiochemotherapy will improve the overall survival by intention to treat for patients with resectable or borderline resectable pancreatic adenocarcinoma through improvement of the resection rate and improvement of the R0 resection rate.

### Study endpoints

The primary endpoint is overall survival by intention to treat. Secondary endpoints include the resection rate, the R0 resection rate, disease-free survival, time to loco-regional recurrence or distant metastases and postoperative complications. A loco-regional failure is any persistent or new sign of tumour in the original tumour location or in the N1 lymph node areas. Secondary endpoints for the patients in the preoperative radiochemotherapy arm also include the toxicity according to Common Terminology Criteria for Adverse Events (CTC-AE) version 4 [42], as well as response to radiochemotherapy according to radiologic Response Evaluation Criteria in Solid Tumors (RECIST) criteria version 1.1 [43] and pathologic response rates after preoperative radiochemotherapy [44].

### Statistical aspects

The median overall survival by intention to treat is estimated to be 11 months in patients treated by explorative laparotomy without preoperative treatment [22, 26, 27]. The study is designed to show a benefit in median overall survival for radiochemotherapy of 6 months (to 17 months), which corresponds to a hazard ratio of 0.651, based on previous studies reporting survival by intention to treat with preoperative radiochemotherapy [19–25].

To achieve 80 % power for the expected median survival difference, taking into account the planned interim analysis and assuming 10 % dropouts, the calculated sample size of the study is 122 patients in each group, adding up to a total of 244 patients, with a total number of 176 events required. The total duration of patient accrual is expected to exceed the initially intended 36 months by about 15 months. The sample size is not separately calculated for both stratification groups (resectable/borderline resectable), but subset analyses for these strata are planned.

A formal interim analysis of all-cause survival will be performed by an independent data monitoring committee at appointed time points to be able to stop the study in case of an unexpectedly high efficacy difference or futility. All analyses will be performed primarily by intention to treat. The stratified log rank test statistics will be used to compare the Kaplan-Meier survival curves of the two randomization groups. The comparison of the time to loco-regional failure and the time to distant metastases between the randomization groups will be analysed by a competing risk model. Subset analyses of the resectable and borderline resectable tumours are planned.

### Study population

Patients meeting the DPCG definitions for resectable or borderline resectable pancreatic adenocarcinoma are eligible. In this study, borderline resectable tumours are tumours with arterial abutment less than 90° contact and/or venous involvement (90–270° contact but without vessel occlusion; Table 1) on CT/MRI. There is currently consensus throughout the Netherlands about these strict criteria. The definition for (borderline) resectable pancreatic tumours differs from those of other countries and the National Comprehensive Cancer Network (NCCN) definition.

### Inclusion criteria

Inclusion criteria are: histologically or cytologically confirmed adenocarcinoma of the pancreas; primarily resectable or borderline resectable tumours (Table 1), with the ability to undergo surgery and radiochemotherapy (WHO ≤1 and normal blood count, leucocytes, platelets, haemoglobin and adequate renal function), with provision of written informed consent.

### Exclusion criteria

Exclusion criteria are: T1 resectable tumours, locally advanced, irresectable tumours or distant metastases; cytologically proven N2 lymph node metastases; carcinoma of the papilla of Vater or distal bile duct; previous active malignancy shorter than 5 years before diagnosis of pancreatic cancer or co-morbidity or previous treatment precluding surgery or radiochemotherapy.

### Randomization

After confirmation of eligibility including written informed consent, patients are randomized, and stratified for resectability (resectable versus borderline resectable) and for the participating institution. Randomization is between arm A: (standard) explorative laparotomy with resection if possible, followed by adjuvant chemotherapy, and arm B: (experimental) preoperative radiochemotherapy after diagnostic laparoscopy, to rule out small liver or peritoneal metastases followed by explorative surgery with resection if possible followed by the remaining adjuvant chemotherapy. The minimum requirements for a successful laparoscopy are visualization of the peritoneum, liver surface, diaphragm and base of the large bowel mesenterium. In both study arms the indications not to continue with a resection will be based upon the finding of distant metastases or the loco-regional extension of the disease, in particular vascular involvement. In case of irresectability or metastasis, such a resection is considered impossible or inappropriate, and it is left to the discretion of the treating surgeon how to proceed with the operation. For instance, palliative bypass surgery may be performed. In the experimental arm, after radiochemotherapy an exploration is performed unless post radiochemotherapy imaging reveals distant metastases or clear-cut loco-regional progression to irresectable disease.

### Treatment

#### Arm A: direct explorative laparotomy

The explorative laparotomy will be performed within 4 weeks after randomization. The indication to continue with a resection will be based upon the absence of distant metastases and the loco-regional extension of the disease, in particular massive vascular involvement. Suspected metastases or invasion of vascular structures, if suspected, must be proven histologically by frozen section. If resection is performed, the standard procedure depending on the location of the cancer is the pylorus-preserving pancreatoduodenectomy (PPPD) with removal of lymph nodes at the right side of the portal vein or a distal pancreatectomy. Both procedures are performed according to a consensus statement by the International Study Group on Pancreatic Surgery [45].

After resection, adjuvant gemcitabine is given. Gemcitabine is administered at 1000 mg/m^2^/dose on days 1, 8, and 15 in six cycles of 28 days (4 weeks) each [25, 38, 46].

#### Arm B: preoperative radiochemotherapy followed by explorative laparotomy

Diagnostic laparoscopy is performed after randomization but prior to the start of radiochemotherapy to rule out peritoneal or small liver metastases that are not visible with imaging. The first chemotherapy cycle has to start not later than 4 weeks after randomization. If needed, preoperative biliary drainage will be performed, preferably with self-expandable metal stents.

A hypofractionated scheme radiotherapy of 15 fractions of 2.4 Gy in 3 weeks will be applied, combined with a course of full dose gemcitabine, 1000 mg/m^2^ on days 1, 8 and 15 followed by one week rest. This course is preceded and followed by a modified course of gemcitabine, 1000 mg/m^2^ on days 1 and 8 followed by one week rest, as described previously [25].

**Fig. 1:**
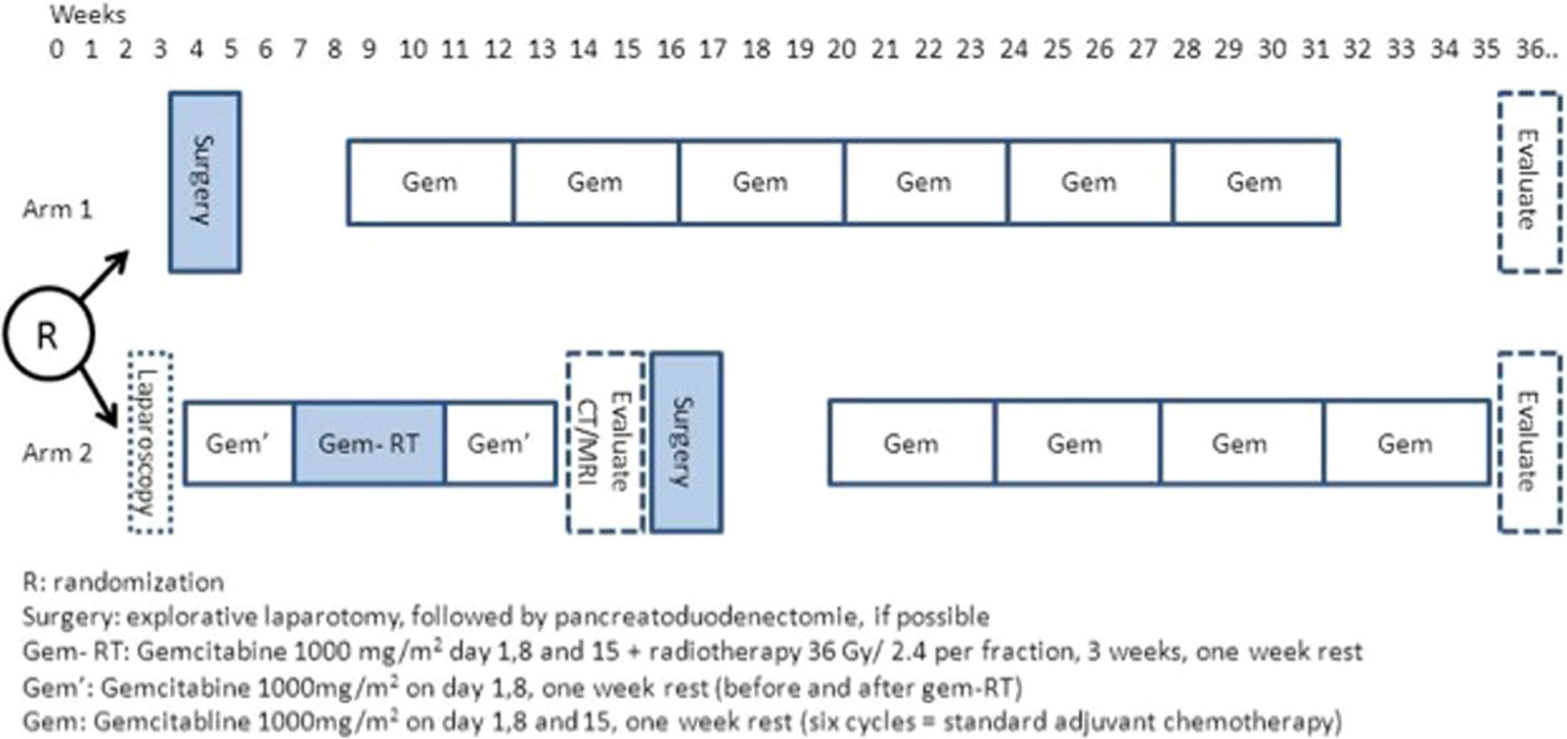
Treatment schedule

The target volumes are delineated on a 4D CT scan with intravenous contrast enhancement. The target volumes include the gross tumour volume (GTV), which includes the pancreatic tumour plus pathologic neighbouring lymph nodes, as described on the diagnostic CT scan. The clinical target volume (CTV) includes the GTV plus possible tumour extension of 5 mm. The internal target volume (ITV) is the sum of the individual defined CTVs in all phases of respiration on the 4D CT scan. Finally, the planning target volume (PTV) is composite, including the ITV plus 10 mm margin. No elective lymph node areas are included in the target volumes. This delineation is done with the diagnostic CT scan (and/or MRI) available in close co-operation between the radiation oncologist and diagnostic radiologist. Scans should be matched when possible with pre-treatment diagnostic CT or MR images if these provide better delineation of the tumour than the dedicated CT scan. At least 95 % of the prescribed dose of 36 Gy in 15 fractions should cover 98 % of the PTV. The preoperative radiochemotherapy schedule is depicted in Fig. 1.

Four weeks after the end of radiochemotherapy a CT scan or MRI is repeated to rule out disease progression by distant metastases and/or overt loco-regional progression and to measure the response according to the RECIST criteria. Thereafter the patient is discussed again by the multidisciplinary team and the exploratory laparotomy is planned, provided that there is no progression. Explorative laparotomy must be performed not earlier than 14 weeks and no later than 18 weeks after randomization. After resection, the remaining gemcitabine is administered at 1000 mg/m^2^/dose on days 1, 8, and 15 in four cycles of 28 days (4 weeks) each to complete a total of seven courses, two of which (courses 1 and 3) are modified in arm A.

### Follow-up

Postoperative care takes place according to the institutions’ routine guidelines. After randomization the patients will be followed up at 6 weeks, 3 months, 6 months and every 6 months thereafter, or more often if the situation of the patient requires so. In both arms there will be follow-up CT scans (or MRIs, at the discretion of the participating institution) at 6, 12, 18 and 24 months, and yearly thereafter.

### Safety

All participating institutions will be monitored for conduct of the trial according to good clinical practice (GCP) standards, at least on a yearly basis [47].

An independent data monitoring committee (IDMC) will monitor the safety of the trial subjects by qualitative analyses of feasibility, accrual rate, and toxicity/morbidity in the first years of the trial, after inclusion of the first 30 patients and thereafter, whenever it is considered appropriate. One formal interim analysis for efficacy or futility is planned after 100 patients have been followed until death or for at least 12 months after inclusion. Serious adverse events will be collected and recorded according to the GCP throughout the study period, defined as from randomization of the first patient through 28 days after the last treatment of the last patient.

### Quality assurance radiotherapy

Every site will be asked to fill out a questionnaire and provide data on a radiation plan of a patient with pancreatic cancer of whom the CT data are provided (a dummy run). The quality assurance team (consisting of at least two experienced radiation oncologists and a physicist) will decide whether the provided plan is adequate, and if not, give recommendations on how to improve it. The results of this dummy run are in preparation for publication.

### Ethics

The study is performed in accordance with the declaration of Helsinki and the Dutch Medical Research Involving Human Subjects Act (WMO) [48, 49]. The protocol has been approved by the Medical Ethical Committee of the Erasmus Medical Centre (MEC-2012-249; date 11-12-2012).

## Discussion

The PREOPANC trial investigates whether the addition of preoperative radiochemotherapy to the standard treatment, consisting of explorative laparotomy (if possible resection via pancreatoduodenectomy or distal pancreatectomy) followed by adjuvant chemotherapy, improves the overall survival (by intention to treat) of patients with resectable or borderline resectable pancreatic cancer (DPCG definition, Table 1). Preoperative radiochemotherapy may improve resection rates as well as R0 resection rates and hence improve overall survival [19–23, 33–38]. A major difficulty in the interpretation of this literature is that most studies are single arm studies that often only report on the subset of patients actually undergoing a resection. This hampers comparison of study results and disables proper analysis of a potential increase in resection rate. From the literature it is suggested that both patients with resectable and borderline resectable tumours may benefit, but it is hard to distinguish if this potential benefit differs between these groups. Hence, the sample size in this study is not calculated for both groups separately. Patients are stratified by resectability status, and subset analyses are planned for all endpoints, possibly leading to observational data on a differential effect.

The treatment schedule in this study consists of full dose gemcitabine (1000 mg/m^2^) combined with radiation, adopted from previous phase II studies [25, 46]. In these studies this radiochemotherapy regimen was well tolerated and safe in patients with pancreatic cancer. It also showed a favourable response rate in the pathologic evaluation after resection [46]. The schedule is based on the rationale that the chemotherapy dose in classical radiochemotherapy schedules was considered too low for the large proportion of early systemic failures in pancreatic cancer. Hence full dose chemotherapy was standard, and a phase I dose-escalating study was performed concerning the dose of radiation, leading to the relatively low dose of radiation of 32 Gy in 15 fractions during the middle course of chemotherapy.

In the experimental arm, a diagnostic laparoscopy is performed before the preoperative radiochemotherapy, to avoid a toxic, yet unnecessary treatment in patients with peritoneal or small liver metastases that are not visible with imaging. Previous studies showed that laparoscopic staging avoided laparotomy in 35–40 % of the patients with pancreatic cancer [50, 51]. All randomized patients who do not undergo a resection for whatever reason are considered a failure for the resection rate endpoint.

The only way to evaluate the role of preoperative radiochemotherapy in improving resection rate, R0 resection rate and thus overall survival is to perform a randomized study, analysing the results by intention to treat [37].

## Trial status

The PREOPANC trial is a Dutch randomized, controlled, multicentre trial, designed to investigate a potential improvement in overall survival for patients with (borderline) resectable pancreatic cancer treated with preoperative radiochemotherapy, followed by explorative laparotomy. The study was opened in April 2013 with two active institutions. At the time of submission of this paper (February 2016) 15 institutions were actively recruiting and 2 pending. A total of 148 patients were accrued in the trial on 6 March 2016.

## Abbreviations

CTV: clinical target volume
DPCG: Dutch Pancreatic Cancer Group
GTV: gross tumour volume
Gy: gray
ITV: internal target volume
mOS: median overall survival
PTV: planning target volume
RECIST: Response Evaluation Criteria in Solid Tumors
